# Blockade of interleukin-6 receptor in the periphery promotes rapid and sustained antidepressant actions: a possible role of gut–microbiota–brain axis

**DOI:** 10.1038/tp.2017.112

**Published:** 2017-05-30

**Authors:** J-c Zhang, W Yao, C Dong, C Yang, Q Ren, M Ma, K Hashimoto

**Affiliations:** 1 Division of Clinical Neuroscience, Chiba University Center for Forensic Mental Health, Chiba, Japan

## Abstract

Depression is a common, severe and chronic psychiatric disease. Although the currently available antidepressants have been used in the treatment of depression, their beneficial effects are limited. Accumulating evidence suggests that pro-inflammatory cytokines such as interleukin-6 (IL-6) have an important role in the pathogenesis of depression. This study was undertaken to examine whether anti-mouse IL-6 receptor antibody (MR16-1) induces antidepressant effects in a social defeat stress model. Intravenous injection of MR16-1 induced rapid-onset and long-lasting antidepressant effects in susceptible mice after social defeat stress through its anti-inflammatory actions. In contrast, intracerebroventricular injection of MR16-1 induced no antidepressant effects in susceptible mice. Furthermore, treatment with MR16-1 could significantly normalize alterations in the expression of synaptic proteins (postsynaptic density protein 95 and α-amino-3-hydroxy-5-methyl-4-isoxazolepropionic acid receptor 1) and dendritic spine density in the brain regions of susceptible mice. Gut microbiota analysis using 16S ribosomal RNA gene sequencing showed that MR16-1 significantly improved decreased *Firmicutes/Bacteroidetes* ratio in susceptible mice. It also significantly improved decreased levels of *Oscillospira* in susceptible mice. These findings suggest that peripheral IL-6 has a key role in the pathogenesis of depression and that the blockade of IL-6 receptor in the periphery might have rapid-onset and long-lasting antidepressant effects by normalizing the altered composition of gut microbiota in susceptible mice after social defeat stress. Therefore, the blockade of IL-6 receptor in the periphery shows promise as a novel therapeutic approach for depressed patients with higher IL-6 blood levels.

## Introduction

Depression is a significant contributor to the global burden of disease and affects people in all communities across the world. It is estimated to affect 350 million people, and almost 1 million lives are lost annually due to suicide. The World Health Organization predicts that depression will be the single leading cause of burden of all health conditions by 2030.^1^ Although inflammation has a central role in the pathogenesis of depression,^2, 3, 4, 5, 6, 7^ the precise mechanisms underlying inflammation-induced depression remain undetermined. Accumulating evidence suggests that interleukin-6 (IL-6), one of the pro-inflammatory cytokines, has an important role in the pathogenesis of depression.^8, 9, 10, 11, 12, 13, 14^ Meta-analyses showed that depressed patients have higher levels of multiple inflammatory markers, including IL-6.^15, 16, 17^ Furthermore, blood levels of IL-6 in patients with suicidality were significantly higher than those in patients without suicidality and healthy control subjects, suggesting that peripheral IL-6 may be associated with suicidal ideation, a core symptom of depression.^11^ Interestingly, we reported that alterations in peripheral, but not brain, IL-6 level might contribute to resilience versus susceptibility to inescapable stress in the rat learned helplessness model.^18^ Taken together, it is likely that peripheral IL-6 might be involved in the pathogenesis of inflammation-induced depression.^12, 13, 18^

The gut–microbiota–brain axis is a complex multi-organ bidirectional signaling system between the microbiota and brain that has a fundamental role in host physiology, homeostasis, development and metabolism.^19, 20, 21^ Accumulating evidence suggests the reproducible and consistent effects of microbial states on mouse behavior, supporting the role of microbiota in behavior.^22, 23, 24^ Recent studies have demonstrated that abnormalities in the composition of gut microbiota might have a causative role in depression.^25, 26, 27^

Considering the key role of IL-6 in depression, the present study investigated whether anti-mouse IL-6 receptor antibody (MR16-1) induces antidepressant-like effects in the social defeat stress model. Next, we examined the role of synaptogenesis in the selected brain regions for the antidepressant actions of MR16-1. Finally, we examined the role of gut microbiota in the antidepressant actions of MR16-1 in the social defeat stress model.

## Materials and methods

### Animals

Male adult C57BL/6 mice (*n*=265), aged 8 weeks (body weight 20–25 g, Japan SLC, Hamamatsu, Japan), and male CD1 mice (*n*=40), aged 14 weeks (body weight 40–45 g, Japan SLC) were used in the experiments. The animals were housed under controlled temperature and 12 h light/dark cycles (lights on between 0700 and 1900 h), with *ad libitum* food and water. The protocol was approved by the Chiba University Institutional Animal Care and Use Committee (permission number: 27–125 and 28–273).

### Intravenous administration of rat MR16-1

Rat MR16-1 (a gift from Chugai Pharmaceutical (Tokyo, Japan)) was used.^28^ On the injection day, the mice were given intravenous injection of MR16-1 (2 mg per 0.2 ml per mouse) or rat IgG isotype control (clone 43414, R&D Systems, Minneapolis, MN, USA).

### Intracerebroventricular administration of MR16-1

**Figure 1:**
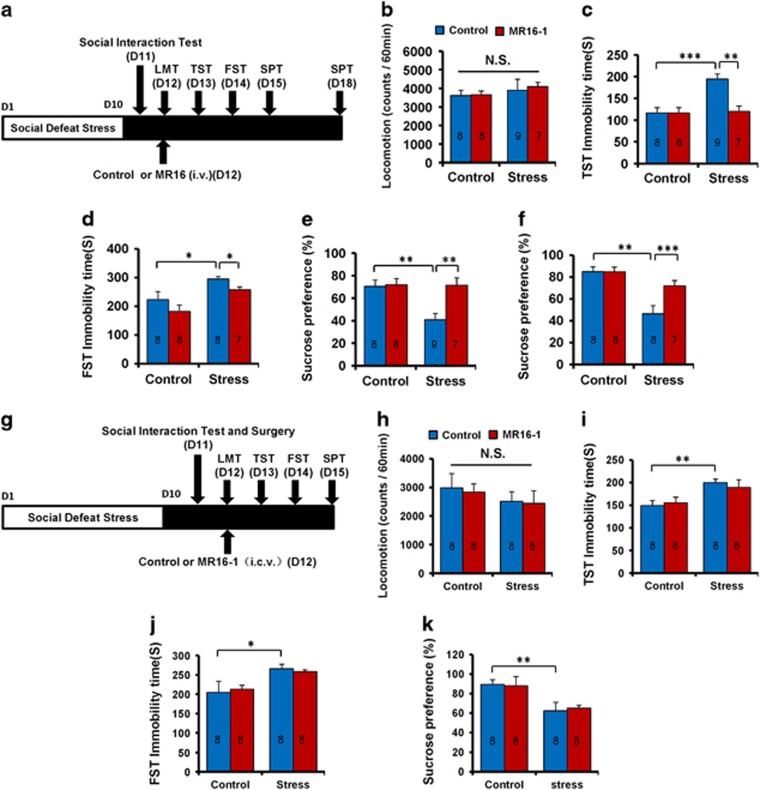
Antidepressant effects of MR16-1 in social defeat stress model. (**a**) Schedule of social defeat stress (10 days), social interaction test, drug treatment (intravenous (i.v.)), and behavioral tests. (**b**) Locomotion test (LMT). (**c**) Tail suspension test (TST). (**d**) Forced swimming test (FST). (**e**) One % sucrose preference test (SPT). (**f**) One % SPT. Two-way analysis of variance (ANOVA) revealed the results (LMT (**b**), Stress: F_1,31_=0.843, *P*=0.366; MR16-1: F_1,31_=0.064, *P*=0.801; interaction: F_1,31_=0.078, *P*=0.782), (TST (**c**), Stress: F_1,31_=11.351, *P*=0.002; MR16-1: F_1,31_=9.425, *P*=0.005; interaction: F_1,31_=9.317, *P*=0.005), (FST (**d**); Stress: F_1,30_=15.389, *P*=0.001; MR16-1: F_1,30_=4.363, *P*=0.046; interaction: F_1,27_=0.008, *P*=0.929), (SPT (**e**); Stress: F_1,31_=6.312, *P*=0.018; MR16-1: F_1,31_=7.259, *P*=0.012; interaction: F_1,31_=6.002, *P*=0.021), (SPT (**f**); Stress: F_1,30_=5.126, *P*=0.032; MR16-1: F_1,30_=5.086, *P*=0.032; interaction: F_1,30_=20.956, *P*<0.001). The values represent the mean±s.e.m. (*n*=7–9). *P<0.05, **P<0.01, ***P<0.001 compared with the control-treated stress group. (**g**) Schedule of social defeat stress (10 days), social interaction test and surgery, drug treatment (intracerebroventricular (i.c.v.)), and behavioral tests. (**h**) LMT. (**i**) TST. (**j**) FST. (**k**) One % SPT. Two-way ANOVA revealed the results (LMT (**h**), Stress: F_1,32_=1.406, *P*=0.245; MR16-1: F_1,32_=0.091, *P*=0.765; interaction: F_1,32_=0.009, *P*=0.926), (TST (**i**), Stress: F_1,31_=12.465, *P*=0.001; MR16-1: F_1,31_=0.014, *P*=0.905; interaction: F_1,31_=0.391, *P*=0.537), (FST (**j**); Stress: F_1,31_=12.149, *P*=0.002; MR16-1: F_1,31_=0.027, *P*=0.871; interaction: F_1,31_=0.476, *P*=0.496), (SPT (**k**), Stress: F_1,31_=12.737, *P*=0.001; MR16-1: F_1,31_=0.008, *P*=0.929; interaction: F_1,31_=0.094, *P*=0.762). The values represent the mean±s.e.m. (*n*=8). *P<0.05 and **P<0.01 compared with the control+stressed group. The number in the column is the number of mice. NS, not significant.

After the social interaction test (day 11), susceptible mice were anesthetized with isoflurane and pentobarbital (5 mg ml^−1^ and 0.15 ml per mouse) and placed in a stereotaxic frame. The cannulas were placed into the lateral ventricles (+0.02 anteroposterior, +1.0 mediolateral, −1.5 dorsoventral).^29^ Twenty-four hours after surgery (day 12 in Figure 1g), MR16-1 (0.01 mg μl^−1^, 0.1 μl min^−1^ for 20 min) or control IgG was injected into susceptible mice.

### Social defeat stress model

Briefly, C57BL/6 mice were exposed to a different CD1 aggressor mouse each day for 10 min for 10 days.^30, 31, 32, 33, 34, 35, 36, 37, 38, 39^ After the social defeat session, the resident CD1 mouse and intruder mouse were housed in one-half of the cage separated by a perforated Plexiglas divider to allow visual, olfactory and auditory contact for the remainder of the 24 h period. Twenty-four hours after the last session, all the mice were housed individually. On day 11, a social avoidance test was performed to identify the subgroups of mice that were susceptible and unsusceptible to social defeat stress. Approximately 70% of mice were susceptible in this study. Only susceptible mice were used in the subsequent experiments.

A social interaction test was performed 1 day (day 11) after the last social defeat session. For this test, an open-field arena (42 × 42 cm) was divided into an interaction zone and two opposing corner zones. A mesh plastic target box (10 × 4.5 cm) was placed in the interaction zone. A test mouse was allowed to roam around the open-field arena for 2.5 min with no social target (CD1 mouse) in the mesh box (denoted as ‘no target’ in figures showing results of social interaction experiments). After this, a novel CD1 mouse was placed in a metal mesh plastic target box in the interaction zone (denoted as ‘target’ in figures showing results of social interaction experiments) and the test mouse was placed back into the open arena for another 2.5 min. Using the stopwatch, the amount of time spent in the interaction zone (defined as the 8 cm wide area surrounding the wire mesh cage) was measured both with and without the social target.^32^

### Behavioral tests

Locomotion: the mice were placed in experimental cages (length × width × height: 560 × 560 × 330 mm).^33, 34, 35, 36, 37, 38, 39, 40^ Locomotor activity of mice was counted using SCANET MV-40 (MELQUEST, Toyama, Japan), and cumulative exercise was recorded for 60 min. The cages were cleaned between the testing sessions. Tail suspension test: the mice were taken from their home cages and a small piece of adhesive tape was placed approximately 2 cm from the tip of their tails. A single hole was punched in the tape and the mice were hung individually on a hook. The immobility time of each mouse was recorded for 10 min. They were considered immobile only when they hung passively and completely motionless. Forced swimming test: the mice were placed individually in a cylinder (diameter: 23 cm; height: 31 cm) containing 15 cm of water, maintained at 23±1°C. They were tested in an automated forced-swim apparatus using SCANET MV-40. Immobility time was calculated from activity time as total active time using the apparatus analysis software. Cumulative immobility time was scored for 6 min during the test. Sucrose preference test: the mice were habituated to a 1% sucrose solution for 48 h before the test day. They were deprived of water and food for 4 h, followed by a preference test spanning 1 h with water and 1% sucrose, delivered from identical bottles. The bottles containing water and sucrose were weighed before and at the end of this period and the sucrose preference (%) was determined.

### Enzyme-linked immunosorbent assay

The blood samples were obtained via a cardiac puncture 1 or 7 days after MR16-1 administration. The blood was centrifuged at 2000 *g* for 20 min to generate serum samples. The serum samples were diluted 10-fold with ELISA (enzyme-linked immunosorbent assay) diluent solution (eBioscience, San Diego, CA, USA). The serum levels of tumor necrosis factor-α (TNF-α), interleukin-1β (IL-1β) and interleukin-6 (IL-6) were measured using a Ready-SET-Go ELISA kit (eBioscience) according to the manufacturer’s instructions.

### Western blot analysis

The brain samples of CA1, CA3 and DG of the hippocampus, prefrontal cortex (PFC) and nucleus accumbens (NAc) from mice were dissected as previously reported.^33, 34, 37, 38, 39, 40^ The tissue samples were homogenized in Laemmli lysis buffer. The aliquots (10 μg) of protein were measured using a DC protein assay kit (Bio-Rad, Hercules, CA, USA) and incubated for 5 min at 95 °C, with an equal volume of 125 mm Tris/HCl, pH 6.8, 20% glycerol, 0.1% bromophenol blue, 10% β-mercaptoethanol and 4% sodium dodecyl sulfate, and subjected to sodium dodecyl sulfate polyacrylamide gel electrophoresis using 10% mini-gels (Mini-PROTEAN TGX Precast Gel; Bio-Rad). The proteins were transferred onto polyvinylidene difluoride membranes using a Trans Blot Mini Cell (Bio-Rad). For immunodetection, the blots were blocked with 2% bovine serum albumin in TBST (TBS+0.1% Tween-20) for 1 h at room temperature and kept with primary antibodies overnight at 4 °C. The following primary antibody was used: postsynaptic density protein 95 (PSD-95; 1 μg ml^−1^ Invitrogen, Carlsbad, CA, USA). The next day, blots were washed three times in TBST and incubated with horseradish peroxidase conjugated anti-rabbit antibody (1:5000) for 1 h, at room temperature. After the final three washes with TBST, bands were detected using enhanced chemiluminescence plus the Western Blotting Detection system (GE Healthcare Bioscience, Tokyo, Japan). The blots then were incubated in the stripping buffer (2% sodium dodecyl sulfate, 100 mm β-mercaptoethanol and 62.5 mmTris-HCl, pH 6.8) for 30 min at 60 °C and then washed three times with TBST. The stripped blots were kept in blocking solution for 1 h and incubated with the primary antibody directed against α-amino-3-hydroxy-5-methyl-4-isoxazolepropionic acid receptor (AMPAR) 1 (GluA1; 1 μg ml^−1^ Abcam, Cambridge, MA, USA) and β-actin. Images were captured with a Fuji LAS3000-mini imaging system (Fujifilm, Tokyo, Japan) and immunoreactive bands were quantified.

### Golgi staining

Golgi staining was performed using the FD Rapid GolgiStain Kit (FD Neuro Technologies, Columbia, MD, USA), following the manufacturer’s instructions.^38, 39, 40^ The animals were deeply anesthetized with isoflurane and pentobarbital, and the brains were removed from the skulls and rinsed in double distilled water. The brains were immersed in the impregnation solution, prepared by mixing equal volumes of solutions A and B overnight, and then stored in a fresh solution for 2 weeks in the dark. The brains were transferred into solution C overnight and then stored in fresh solution at 4 °C for 1 week in the dark. Coronal brain sections (100 μm thickness) were cut on a cryostat (3050S, Leica Microsystems, Wetzlar, Germany), with the chamber temperature set at −20 °C. Each section was mounted in solution C on saline-coated microscope slides. After absorption of excess solution, the sections were dried naturally at room temperature. The dried sections were processed following the manufacturer’s instructions. Briefly, images of dendrites within CA1, CA3 and DG of the hippocampus, PFC and NAc were captured using a × 100 objective with a Keyence BZ-9000 Generation II microscope (Osaka, Japan). The spines were counted along the CA1, CA3, DG, PFC and NAc dendrites starting from their points of origin from the primary dendrite, as previously reported.^38, 39, 40, 41, 42, 43^ For spine density measurements, all clearly evaluable areas containing 50–100 μm of secondary dendrites from each imaged neuron were used. To determine relative spine density, the spines on multiple dendritic branches from a single neuron were counted to obtain an average spine number per 10 μm. For spine number measurements, only spines that emerged perpendicular to the dendritic shaft were counted. Three neurons per section, three sections per animal and six animals were analyzed. The average value for each region in each individual was obtained. These individual averages were then combined to yield a grand average for each region.

### 16S rRNA analysis of fecal samples

The fecal samples were collected 7 days after MR16-1 (or control IgG) administration and placed in 1.5 ml tubes, snap-frozen on dry ice and stored at −80 °C. The 16 S rRNA analysis of fecal samples was performed at Takara Bio (Shiga, Japan). The DNA extraction was performed using the MoBio Powerlyzer Powersoil DNA Isolation Kit (MoBio Laboratories, Carlsbad, CA, USA). The V4 hypervariable region of the bacterial 16S rRNA gene was amplified from the fecal DNA extracts using modified universal bacterial primer pairs 515F (5′-TCGTCGGCAGCGTCAGATGTGTATAAGAGACAGGTGCCAGCMGCCGCGGTAA-3′) and 806R (5′-GTCTCGTGGGCTCGGAGATGTGTATAAGAGACAGGGACTACHVGGGTWTCTAAT-3′) with Illumina adaptor overhang sequences. Amplicons were generated, cleaned, indexed and sequenced according to the Illumina MiSeq 16S Metagenomic Sequencing Library Preparation protocol (http://support.illumina.com/downloads/16s_metagenomic_sequencing_library_preparation.html) with certain modifications.

Sequencing data were combined and sample identification assigned to multiplexed reads using the MOTHUR software environment.^44^ The data were denoised; low quality sequences, pyrosequencing errors, and chimeras were removed, and then sequences were clustered into operational taxonomic units (OTUs) at 97% identity using the CD-HIT-OTU pipeline (available from http://eeizhong-lab.ucsd.edu/cd-hit-otu).^45^ OTUs containing fewer than four reads per individual diet/animal combination were excluded due to the likelihood of there being a sequencing artifact. The samples were normalized by randomly resampling sequences used to the lowest number of sequences per sample (each diet/animal combination) using Daisychopper (http://www.festinalente.me/bioinf/). Taxonomic classification of OTUs was conducted using the Ribosomal Database Project Classifier.^46^

### Statistical analysis

The data are shown as mean±s.e.m. Analysis was performed using PASW Statistics 20 (formerly SPSS statistics; SPSS, Tokyo, Japan). Comparisons between groups were performed using one-way analysis of variance followed by the *post hoc* least significant difference test or two-way analysis of variance; when appropriate, *post hoc* comparisons were performed using the unpaired *t*-test. *P*-values <0.05 were considered statistically significant.

## Results

### Antidepressant effects of MR16-1 in the social defeat stress model

To examine the antidepressant effects of MR16-1 in the social defeat stress model, intravenous injection of MR16-1 or control was administered 60 min before the locomotion test (Figure 1a). No effect was observed in spontaneous locomotion in the four groups (Figure 1b). In the tail suspension test, MR16-1 significantly attenuated the increased immobility time observed in susceptible mice after social defeat stress (Figure 1c). In the forced swimming test, there was no significant interaction (Figure 1d). In the 1% sucrose preference test, MR16-1 significantly attenuated decreased sucrose preference in susceptible mice 3 and 6 days after a single dose (Figures 1e and f). The results suggest that intravenous injection of MR16-1 showed rapid and sustained antidepressant effects in a social defeat stress model.

It was reported that peripheral, but not brain, IL-6 might be involved in the depression-like phenotypes in rodents.^8, 13, 18^ Therefore, we examined whether intracerebroventricular injection of MR16-1 showed antidepressant effects in the social defeat stress model (Figure 1g). No effect was observed in spontaneous locomotion in the four groups (Figure 1h). In the tail suspension test and forced swimming test, intracerebroventricular injection of MR16-1 did not attenuate the increased immobility time of susceptible mice (Figures 1i and j). Moreover, MR16-1 did not affect the decreased sucrose preference of susceptible mice (Figure 1k). These results suggest that intracerebroventricular injection of MR16-1 did not show antidepressant effects in the social defeat stress model. Collectively, it is likely that the blockade of the IL-6 receptor in the periphery may be involved in the antidepressant effects of MR16-1.

### Anti-inflammatory effects of MR16-1 in the serum of susceptible mice after social defeat stress

**Figure 2:**
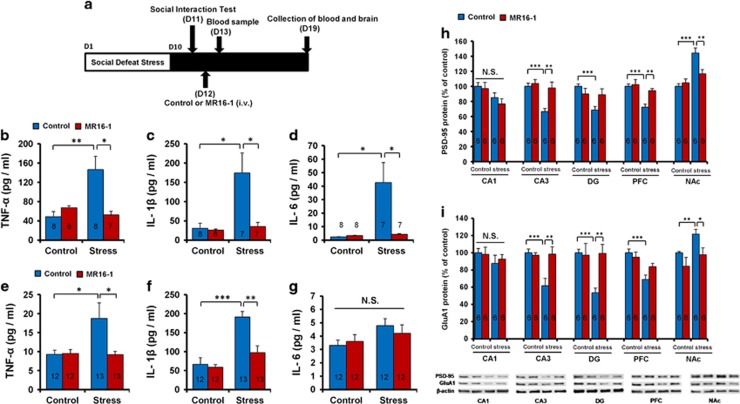
Anti-inflammatory effects of MR16-1 in serum and the expression of synaptic proteins in the brain of susceptible mice after social defeat stress. (**a**) Schedule of social defeat stress (10 days), social interaction test, drug treatment and sample collection. Samples of blood and brain were collected 1 day and 7 days after intravenous (i.v.) injection of MR16-1. Serum levels of tumor necrosis factor (TNF)-α, interleukin (IL)-1β and IL-6 were measured using enzyme-linked immunosorbent assay (ELISA). The levels of PSD-95 and GluA1 in the brain regions were measured using western blot. (**b**–**d**) Serum levels of TNF-α, IL-1β and IL-6 on day 13 (1 day after injection). Two-way analysis of variance (ANOVA) revealed the results (TNF-α (**b**), Stress: F_1,30_=5.541, *P*=0.026; MR16-1: F_1,30_=4.608, *P*=0.041; interaction: F_1,30_=10.326, *P*=0.003), (IL-1β (**c**), Stress: F_1,29_=9.026, *P*=0.006; MR16-1: F_1,30_=7.935, *P*=0.009; interaction: F_1,30_=6.938, *P*=0.014), (IL-6 (**d**), Stress: F_1,30_=7.868, *P*=0.009; MR16-1: F_1,30_=6.487, *P*=0.017; interaction: F_1,30_=7.179, *P*=0.013). Data are shown as the mean±s.e.m. (*n*=7 or 8). *P<0.05 and **P<0.01 compared with the control-treated stress group. (**e**–**g**) Serum levels of TNF-α, IL-1β and IL-6 on day 19 (7 days after injection). Two-way ANOVA revealed the results (TNF-α (**e**), Stress: F_1,47_=4.192, *P*=0.047; MR16-1: F_1,47_=4.301, *P*=0.044; interaction: F_1,47_=4.744, *P*=0.035), (IL-1β (**f**), Stress: F_1,47_=29.068, *P*<0.001; MR16-1: F_1,47_=11.296, *P*=0.002; interaction: F_1,47_=8.032, *P*=0.007), (IL-6 (**g**), Stress: F_1,45_=4.532, *P*=0.039; MR16-1: F_1,45_=0.006, *P*=0.939; interaction: F_1,45_=0.405, *P*=0.528). Data are shown as the mean±s.e.m. (*n*=12 or 13). *P<0.05, **P<0.01, ***P<0.001 compared with the control-treated stressed group. (**h** and **i**) Western blot of PSD-95 and GluA1 in the CA1, CA3 and DG of hippocampus, prefrontal cortex (PFC) and nucleus accumbens (NAc). (**h**) Two-way ANOVA revealed the results (CA1, Stress: F_1,22_=3.256, *P*=0.088; MR16-1: F_1,22_=1.414, *P*=0.250; interaction: F_1,22_=0.536, *P*=0.474), (CA3, Stress: F_1,22_=11.880, *P*=0.003; MR16-1: F_1,22_=9.458, *P*=0.006; interaction: F_1,22_=5.788, *P*=0.026), (DG, Stress: F_1,21_=6.180, *P*=0.023; MR16-1: F_1,21_=0.599, *P*=0.449; interaction: F_1,21_=5.523, *P*=0.030), (PFC, Stress: F_1,22_=15.933, *P*=0.001, MR16-1: F_1,22_=7.278, *P*=0.014; interaction: F_1,22_=4.772, *P*=0.042), (NAc, Stress: F_1,22_=26.386, *P*=0.001; MR16-1: F_1,22_=4.429, *P*=0.049; interaction: F_1,22_=8.705, *P*=0.008). (**i**) Two-way ANOVA revealed the results (CA1, Stress: F_1,22_=1.442, *P*=0.245; MR16-1: F_1,22_=0.043, *P*=0.839; interaction: F_1,22_=0.220, *P*=0.645), (CA3, Stress: F_1,22_=7.600, *P*=0.013; MR16-1: F_1,22_=6.257, *P*=0.022; interaction: F_1,22_=8.696, *P*=0.008), (DG, Stress: F_1,22_=5.075, *P*=0.037; MR16-1: F_1,22_=4.731, *P*=0.043; interaction: F_1,22_=6.105, *P*=0.024), (PFC, Stress: F_1,22_=18.398, *P*<0.001; MR16-1: F_1,22_=0.979, *P*=0.335; interaction: F_1,22_=4.223, *P*=0.054), (NAc, Stress: F_1,22_=5.590, *P*=0.029; MR16-1: F_1,22_=7.094, *P*=0.015; interaction: F_1,22_=0.293, *P*=0.595). The values are expressed as percentages relative to those in the control mice. The values represent the mean±s.e.m. (*n*=5 or 6). *P<0.05, **P<0.01, ***P<0.001 compared with the control-treated stress group. The number in the column is the number of mice. NS, not significant.

To examine the effects of MR16-1 on serum levels of pro-inflammatory cytokines in susceptible mice after social defeat stress, blood samples were collected 1 and 7 days after intravenous injection (Figure 2a). Serum levels of TNF-α, IL-1β and IL-6 in susceptible mice were significantly higher than those in the control group 1 day after injection (Figures 2b–d). MR16-1 significantly attenuated the increased levels of TNF-α, IL-1β and IL-6 in susceptible mice 1 day after injection (Figures 2b–d). Furthermore, MR16-1 significantly attenuated the increased levels of TNF-α and IL-1β in susceptible mice 7 days after injection (Figures 2e and f). However, there were no changes of serum IL-6 levels among the four groups 7 days after injection (Figure 2g).

### Levels of PSD-95 and GluA1 in the brain regions after a single injection of MR16-1

We performed western blot of the synaptogenesis markers, PSD-95 and GluA1 in the brain regions, which were collected 7 days after intravenous injection of MR16-1 or control. Susceptible mice showed decreased protein levels of PSD-95 in the hippocampus (CA3 and DG) and PFC, whereas they showed increased levels of PSD-95 in the NAc (Figure 2h). MR16-1 significantly attenuated alterations in the levels of PSD-95 in the CA3, PFC and NAc (Figure 2h). Furthermore, susceptible mice showed decreased protein levels of GluA1 in the hippocampus (CA3 and DG), whereas they showed increased levels of GluA1 in the NAc (Figure 2i). MR16-1 significantly attenuated alterations in the levels of GluA1 in the CA3, DG and NAc (Figure 2i). Collectively, MR16-1 could normalize alterations in the synaptic proteins (PSD-95 and GluA1) in the brain regions of susceptible mice.

### Effect of MR16-1 on alterations in the dendritic spine density in the brain regions of susceptible mice after social defeat stress

**Figure 3:**
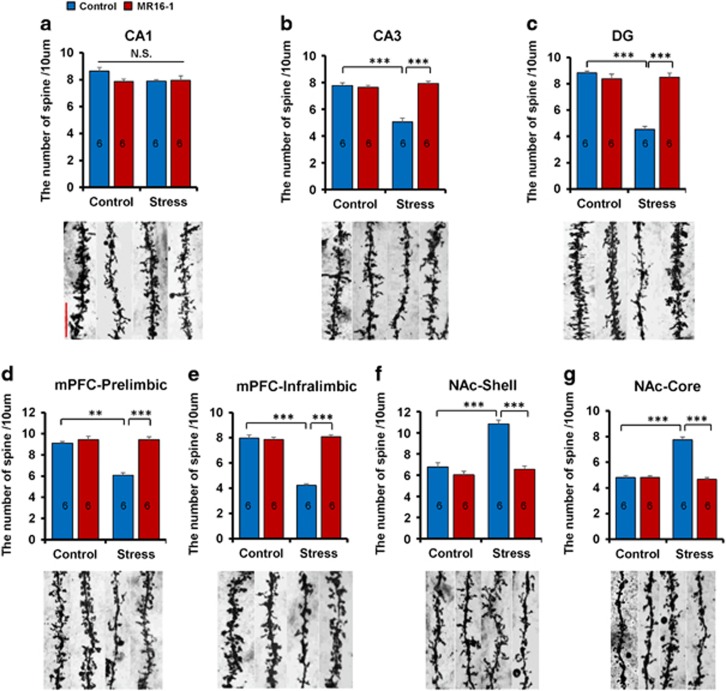
Effects of MR16-1 on alterations in the spine density of brain regions of susceptible mice after social defeat stress. (**a**–**g**) Representative photomicrographs of Golgi-Cox stained pyramidal neurons in the CA1, CA3 and DG of the hippocampus, prelimbic and infralimbic of medial prefrontal cortex (PFC), nucleus accumbens (NAc) shell and NAc core from animals of each group. Scale bar, 10 μm. Two-way analysis of variance (ANOVA) revealed the following results: (CA1 (**a**), Stress: F_1,23_=2.949, *P*=0.101; MR16-1: F_1,23_=2.215, *P*=0.152; interaction: F_1,23_=1.887, *P*=0.185), (CA3 (**b**), Stress: F_1,23_=34.883, *P*<0.001; MR16-1: F_1,23_=43.261, *P*<0.001; interaction: F_1,23_=52.541, *P*<0.001), (DG (**c**), Stress: F_1,23_=60.089, *P*<0.001; MR16-1: F_1,23_=42.512, *P*<0.001; interaction: F_1,23_=66.634, *P*<0.001), (mPFC PrL (**d**), Stress: F_1,23_=28.073, *P*<0.001; MR16-1: F_1,23_=41.098, *P*<0.001; interaction: F_1,23_=28.073, *P*<0.001), (mPFC IL (**e**), Stress: F_1,23_=98.468, *P*<0.001; MR16-1: F_1,23_=111.264, *P*<0.001; interaction: F_1,23_=124.841, *P*<0.001), (NAc shell (**f**), Stress: F_1,23_=29.435, *P*<0.001; MR16-1: F_1,23_=36.049, *P*<0.001; interaction: F_1,23_=16.416, *P*<0.001), (NAc core (**g**), Stress: F_1,23_=72.553, *P*<0.001; MR16-1: F_1,23_=87.632, *P*<0.001; interaction: F_1,23_=87.632, *P*<0.001). The values represent the mean±s.e.m. (*n*=6). **P<0.01, ***P<0.001 compared with the control-treated stress group. The number in the column is the number of mice. NS, not significant.

Alterations in the dendritic length and spine density in the hippocampus, PFC and NAc have an important role in the pathogenesis of depression, and antidepressant treatment can block or reverse these changes.^41, 42, 43, 47, 48, 49^ Susceptible mice showed a decreased spine density in the hippocampus (CA3 and DG) and medial PFC (prelimbic (PrL) and infralimbic (IL) regions), whereas these mice had an increased spine density in the NAc shell and core (Figures 3a–g). Treatment with MR16-1 significantly attenuated the decreased spine density in the CA3, DG and mPFC (PrL and IL) of susceptible mice (Figures 3a–e). In contrast, treatment with MR16-1 significantly attenuated increased spine density in the NAc shell and core of susceptible mice (Figures 3f and g). These results suggest that MR16-1 could induce antidepressant-like effects by normalizing alterations in the spine density in these brain regions of susceptible mice after social defeat stress.

### Antidepressant effects of MR16-1 by normalizing the altered composition of the gut microbiome

**Figure 4:**
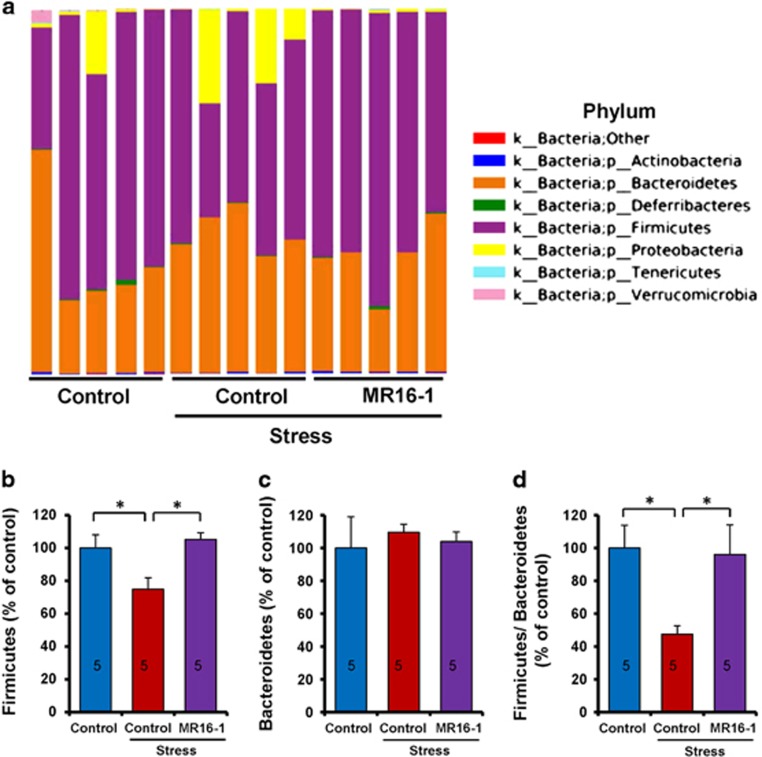
Effects of MR16-1 on alterations in the composition of gut microbiome in phylum of susceptible mice after social defeat stress. (**a**) Phylum. (**b**) Levels of *Firmicutes*. (**c**) Levels of *Bacteroidetes*. (**d**) Ratio of *Firmicutes* to *Bacteroidetes*. One-way analysis of variance (ANOVA) revealed the results (*Firmicutes* (**b**), F_1,14_=5.227, *P*=0.023), (*Bacteroidetes* (**c**), F_1,14_=0.137, *P*=0.873), (*Firmicutes*/*Bacteroidetes* ratio (**d**); F_1,14_=3.964, *P*=0.048). Data are shown as the mean±s.e.m. (*n*=5). *P<0.05 compared with the control-treated stress group. The number in the column is the number of mice. NS, not significant.

For the gut microbiome analysis using 16S ribosomal RNA gene sequencing, fecal samples were collected 7 days after intravenous injection of MR16-1 or control. Susceptible mice showed decreased levels of *Firmicutes* and decreased *Firmicutes/Bacteroidetes* ratio at the phylum level (Figures 4a–d). MR16-1 significantly attenuated a decreased level of *Firmicutes* and decreased *Firmicutes/Bacteroidetes* ratio in susceptible mice after social defeat stress (Figures 4a–d).

**Figure 5:**
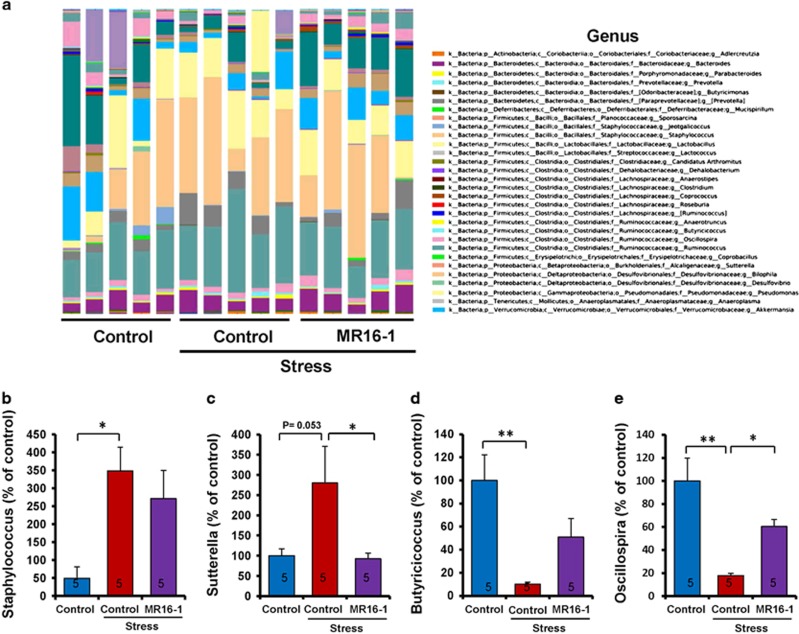
Effects of MR16-1 on alterations in the composition of gut microbiome in genus of susceptible mice after social defeat stress. (**a**) Genus. (**b**) Levels of *Staphylococcus*. (**c**) Levels of *Sutterella*. (**d**) Levels of *Butyricicoccus*. (**e**) Levels of *Oscillospira*. One-way analysis of variance (ANOVA) revealed the results (*Staphylococcus* (**b**), F_1,14_=4.104, *P*=0.044), (*Sutterella* (**c**), F_1,14_=4.505, *P*=0.037), (*Butyricicoccus* (**d**), F_1,14_=6.727, *P*=0.011), (*Oscillospira* (**e**), F_1,14_=9.762, *P*=0.003). Data are shown as the mean±s.e.m. (*n*=5). *P<0.05 and **P<0.01 compared with the control-treated stress group. The number in the column is the number of mice. NS, not significant.

At the genus level, susceptible mice showed increased levels of *Staphylococcus* and decreased levels of *Butyricicoccus* and *Oscillospira* (Figures 5a, b, d and e). Furthermore, susceptible mice revealed a nonsignificant trend toward an increased level of *Sutterella* (Figures 5a and c). MR16-1 significantly improved the increased levels of *Sutterella* and decreased levels of *Oscillospira* in susceptible mice (Figures 5c and e). In addition, MR16-1 nonsignificantly tended to alter the levels of *Staphylococcus* and *Butyricicoccus* (Figures 5b and d).

## Discussion

The major findings of this study are that peripheral, but not brain, IL-6 has an important role in the depression-like phenotype after social defeat stress and that gut microbiota may have a role in the antidepressant effects of anti-IL-6 receptor MR16-1. First, intravenous injection of MR16-1 showed rapid and long-lasting antidepressant effects in the social defeat stress model, although intracerebroventricular injection of MR16-1 did not show antidepressant-like effects in the same model. Second, MR16-1 could attenuate alterations in the synaptic proteins (PSD-95 and GluA1) and dendritic spine density in the brain regions of susceptible mice after social defeat stress. Third, MR16-1 could normalize alterations in the gut microbiota composition in susceptible mice after social defeat stress. These findings suggest that increases in the pro-inflammatory cytokines and altered composition of gut microbiota induced by social defeat stress might have an important role in the pathogenesis of depression and that the gut–microbiota–brain axis may be implicated in the rapid-onset and long-lasting antidepressant actions of anti-IL-6 receptor antibody. The blockade of IL-6 receptor by the humanized anti-IL-6 receptor antibody tocilizumab has been used in the treatment of rheumatoid arthritis,^50^ and a human anti-IL-6 monoclonal antibody, sirukumab, has been under evaluation in patients, including those with rheumatoid arthritis. Therefore, it is likely that a humanized anti-IL-6 receptor antibody (for example, tocilizumab) or a human anti-IL-6 monoclonal antibody (for example, sirukumab) would be potential therapeutic drugs for the blockade of IL-6 signaling in depressed patients with higher blood levels of IL-6.

Social defeat stress has been reported to increase IL-6 release in the serum of patients who subsequently developed a depression-like phenotype.^8^ In addition, serum levels of IL-6 strongly correlated with social interaction behavior following repeated social defeat stress. Stress-susceptible bone marrow chimeras revealed increased social avoidance behavior after exposure to either sub-threshold repeated social defeat stress, or a purely emotional stressor, termed witness defeat.^8^ We also reported that alterations in the peripheral, but not brain, IL-6 might contribute to resilience versus susceptibility to inescapable stress in the rat learned helplessness model.^18^ These results suggest that peripheral, but not brain, IL-6 might be involved in the depression-like phenotype in rodents.^13, 14, 18^ Thus, it is unlikely that the absence of beneficial effects with intracerebroventricular administration may be related to a lack of drug penetration into the parenchymal brain tissues implicated in depression. Collectively, it is likely that the IL-6 receptor antibody MR16-1 showed rapid and long-lasting antidepressant effects in the social defeat stress model by inhibiting IL-6 receptor in the periphery. In addition, IL-6 knockout mice showed resilience to stress-induced development of depression-like behaviors,^51^ suggesting the role of IL-6 in depression.

Accumulating evidence suggests that abnormalities in the gut microbiota composition have a causative role in the pathogenesis of depression.^20, 21, 25, 26, 27, 52^ At the phylum level, *Firmicutes* and *Bacteroidetes* constitute the largest portion of the mouse and human gut microbiome. The *Firmicutes/Bacteroidetes* ratio is shown to be of significant relevance in signaling human gut microbiota status.^53, 54^ For example, obesity is associated with changes in the relative abundance of these two dominant bacterial divisions.^53, 54^ Chronic restraint stress in mice caused a nonsignificant trend toward increased *Firmicutes/Bacteroidetes* ratio, and the anti-inflammatory drug minocycline attenuated increased *Firmicutes/Bacteroidetes* ratio.^26^ Furthermore, increased *Firmicutes/Bacteroidetes* ratio was reported in patients with irritable bowel syndrome.^55^ Thus, increased *Firmicutes/Bacteroidetes* ratio, caused by the increase of *Firmicutes* or decrease of *Bacteroidetes*, has been widely considered a signature of gut dysbiosis. Conversely, a decreased *Firmicutes/Bacteroidetes* ratio was reported to be related to weight loss.^54^ Moreover, the *Firmicutes/Bacteroidetes* ratio is also linked to overall changes in bacterial profiles at different stages of life.^56^ A recent study showed that syringaresinol (a polyphenolic lignan) significantly improved decreased *Firmicutes/Bacteroidetes* ratio in middle-aged mice, suggesting that syringaresinol may rejuvenate the immune system through modulation of gut integrity and microbiota diversity as well as composition in middle-aged mice.^57^ In this study, we found decreased number of *Firmicutes* and decreased *Firmicutes/Bacteroidetes* ratio in susceptible mice after social defeat stress, suggesting that a decreased *Firmicutes/Bacteroidetes* ratio might have a role in the depression-like phenotype. Interestingly, MR16-1 could attenuate the decreased number of *Firmicutes* and decreased *Firmicutes/Bacteroidetes* ratio in susceptible mice. Inflammatory cytokines such as IL-6 and IL-1β in the gut microbiota have a role in inflammation diseases.^58^ Collectively, it is likely that MR16-1 may show antidepressant-like effects by normalizing abnormalities in *Firmicutes* (or *Firmicutes/Bacteroidetes* ratio) through the modulation of the immune system. Nonetheless, further detailed studies underlying the role of gut microbiota in the antidepressant actions of MR16-1 are needed.

At the genus level, *Staphylococcus* and *Sutterella* are genera of gram-positive and gram-negative bacteria, respectively. An increased number of *Sutterella* was reported in the feces of children with autism spectrum disorder, suggesting an imbalance in the gut microbiota in children with autism spectrum disorder.^59^ We found increased numbers of *Staphylococcus* and *Sutterella* in susceptible mice after social defeat stress. Although it is not yet evident what the consequences of increased number of fecal *Staphylococcus* (or *Sutterella*) indicates, it is possible that, under stress conditions, these bacteria may have a role in the depression-like phenotype through infection-induced inflammation. Interestingly, we found that MR16-1 could attenuate the increased numbers of *Staphylococcus* and *Sutterella* in susceptible mice after social defeat stress.

Butylate has a key role in maintaining gut health by preventing cell proliferation, suppressing inflammation and providing energy to enterocytes.^60^ *Butyricicoccus* is a butylate-producing clostridial cluster IV genus whose numbers are reduced in the stool of ulcerative colitis patients.^61, 62^ *Oscillospira*, the clostridial cluster IV of the Firmicutes phylum, also produce butylate. *Oscillospira* is negatively associated with obesity and inflammatory bowel diseases.^63, 64^ In this study, we found that the numbers of *Butyricicoccus* and *Oscillospira* decreased in susceptible mice after social defeat stress and that MR16-1 improved decreased numbers of *Butyricicoccus* and *Oscillospira* in these mice. Interestingly, decreased numbers of fecal *Oscillospira* were detected in patients with depression,^25^ suggesting that alterations in *Oscillospira* number may be involved in the pathogenesis of depression. In addition, sodium butylate, a histone deacetylase inhibitor, showed antidepressant effects in animal models of depression,^65^ suggesting that butylate can be used for treating depression. Considering the role of *Butyricicoccus* and *Oscillospira* in the production of butylate, it is likely that decreased numbers of these bacteria may have a role in the pathogenesis of depression. Reconditioning of the gut microbiota through direct supplementation with beneficial bacteria or by indirect stimulation of colonization and proliferation of beneficial bacteria could have a protective role in inflammation-induced depression. However, detailed studies underlying the role of *Butyricicoccus* and *Oscillospira* in the pathogenesis of depression are needed.

Growing evidence has suggested that alterations in the synaptic proteins (for instance, PSD-95 and GluA1) and dendritic spine density after social defeat stress have a role in the depression-like phenotype and that the recovery of synaptogenesis by antidepressants has a key role in their antidepressant actions.^41, 42, 43, 47, 48, 49^ Intravenous injection of MR16-1 showed a robust antidepressant effect by normalizing decreased levels of PSD-95 and GluA1 in the hippocampus and PFC and increased levels of PSD-95 and GluA1 in the NAc. The rapid-onset and long-lasting antidepressant effects of MR16-1 are similar to those of ketamine (or *R*-ketamine) in the social defeat stress model.^33, 34, 35, 42^

A recent meta-analysis demonstrated higher cerebrospinal fluid levels of IL-6 in depressed patients compared with those in the control subjects.^66^ In this study, we did not find antidepressant effects of MR16-1 after intracerebroventricular injection. Although we did not measure cerebrospinal fluid (or brain) levels of IL-6 in susceptible mice after intracerebroventricular infusion of MR16-1, it is unlikely that brain IL-6 may have a key role in depression-like phenotypes in rodents. Nonetheless, further studies on the role of IL-6 in the brain and periphery will be needed.

Accumulating evidence suggests that immunomodulation by microbiota is an important pathway that orchestrates the gut–microbiota–brain axis.^21^ Alterations in immune homeostasis due to host–microbiota interactions can lead to changes in brain function through the hypothalamus–pituitary–adrenal axis, a neuroendocrine, stress-sensing system that could be activated by pro-inflammatory cytokines.^21^ In this study, we found that the blockade of the IL-6 receptor in the periphery may exert robust antidepressant effects in rodents through immunomodulation by the gut microbiota. Although the precise mechanisms by which the gut microbiota influence brain function remain unclear, there appears to be gut–microbiota–brain axis. Considering the role of the gut microbiota in immunomodulation, it is likely that gut–microbiota–brain communication may have a role in robust antidepressant actions of anti-IL-6 receptor therapy. Future studies using immunodeficient mice will be necessary to confirm the role of the immune system in the gut–microbiota–brain axis in depression.

In conclusion, these findings suggest that increased peripheral IL-6 signaling and altered composition of the gut microbiota may have an important role in the pathogenesis of depression and that IL-6 receptor antibody MR16-1 showed rapid-onset and long-lasting antidepressant effects through the modulation of the immune system. Therefore, it is likely that anti-IL-6 receptor therapy would be a novel therapeutic approach for depressed patients with higher blood levels of IL-6.
